# Purpura

**Published:** 1910-06-18

**Authors:** 


### Dermatology.

### PURPURA.

Purpura is the name given to the rash which is produced by hæmorrhages into the skin. It may occur as a symptom of many diseases. It is seen in scurvy, in hæmophilia, and in cerebro-spinal meningitis. As an occasional symptom it may occur in exanthematic fevers, as measles, smallpox, or scarlet-fever; in patients suffering from cancer, in cases of renal, hepatic, or cardiac disease, and in tuberculosis. Purpura arising under these conditions has been called symptomatic purpura.

Then there are purpuric eruptions which are associated with the ingestion of some poison, such as snake-venom, phosphorus, potassium iodide, and anti-diphtheritic serum. Finally, there is a group of purpuras in which no definite poisoning can be traced, and in which the eruption is not a mere accompaniment of some well-known disease. These have been called idiopathic pupuras.

All that is known as to the causation of purpura points to its origin as a result of some toxæmic condition.

In the presence of a purpuric eruption we may think first of all of scurvy, of hæmophilia, and of the possibility of potassium iodide having been administered. We must examine the heart and the urine and look for any evidence of hepatic disease or of cancer. The possibility of an exanthematic fever, of tuberculosis, or of cerebro-spinal meningitis must also occur to us. Many of these causes can, of course, be excluded at once, but they should run through the mind before finally putting the case into the group of idiopathic purpuras.

Having decided that the purpura is not merely a symptom of one or other of these affections, we shall be able probably to place it into one of the following clinical groups, namely:—

| | |
|---|---|
| (1) Those unassociated with fever or with joint pains | Purpura simplex.<br>Purpura hæmorrhagica. |
| (2) Those accompanied by joint pains or by abdominal pains, or both, and in which the eruption is of an erythematous type. | Purpura rheumatica.<br>Henoch's purpura. |
| (3) Those accompanied by fever ... | Infective purpuras. |

*Purpura simplex*, and purpura hæmorrhagica are virtually of the same class, differing only in that hæmorrhages take place from mucous membranes in the latter and not in the former, though there is no hard-and-fast line to be drawn between them, for a simple purpura will sometimes become "hæmorrhagic." In these cases the patient is often pale and unhealthy looking, though generally without any more definite symptoms of ill-health, and there is no rise of temperature and there are no pains in the joints or abdomen. In fact the purpura and the hæmorrhages from mucous membranes appear to constitute the whole complaint. The duration of the eruption varies from several weeks to many months, and cases occur in which the eruption is present on and off for years. Most of these cases do well if put to bed and fed well, and, generally, no other treatment is necessary, unless severe hæmorrhages occur, when the most effectual remedy is turpentine in large doses. It is best given with castor oil, the dose being from ʒij. to ℥j. of oil of turpentine, with an equal part of castor oil, each morning for two or three days, the dose according to age.

*Purpura rheumatica* (Schönlein's purpura) and Henoch's purpura are associated with pains in the joints, and the rash is not merely that produced by hæmorrhage into the skin, but is preceded by an erythematous eruption such as occurs in erythema multiforme, the hæmorrhage taking place into the erythematous patches. Such cases have been called peliosis rheumatica, although they have no relation to true rheumatic fever; they are possibly really a form of erythema multiforme. Closely allied to these cases are those described by Henoch and generally called "Henoch's purpura." In these cases there are not only joint pains but also abdominal pains, often accompanied by hæmatemesis and passage of blood by the bowel. This affection is most often seen in children, and may be mistaken for acute intussusception. In the treatment of these cases bed is essential, and the diet should be a light one. Cold applications may be used to the abdomen when abdominal symptoms are present, and small doses of opium may be given to relieve pain. The severe cases may be fatal; the milder always recover, though relapses are common.

Infective or febrile purpuras are, on the whole, of a more serious nature than other forms of purpura, for they are due to microbic infections. They are often accompanied by high fever, with a rapidly fatal issue. Various micro-organisms have been cultivated from the blood of these patients, that most frequently found being the streptococcus. The treatment of these cases can only be symptomatic.

